# Artificial neural networks improve early outcome prediction and risk classification in out-of-hospital cardiac arrest patients admitted to intensive care

**DOI:** 10.1186/s13054-020-03103-1

**Published:** 2020-07-30

**Authors:** Jesper Johnsson, Ola Björnsson, Peder Andersson, Andreas Jakobsson, Tobias Cronberg, Gisela Lilja, Hans Friberg, Christian Hassager, Jesper Kjaergard, Matt Wise, Niklas Nielsen, Attila Frigyesi

**Affiliations:** 1 Department of Clinical Sciences Lund, Anesthesia & Intensive Care, Helsingborg Hospital, Lund University, Helsingborg, Sweden; 2 Department of Anaesthesiology and Intensive Care, Helsingborg Hospital, Charlotte Yléns Gata 10, SE-251 87 Helsingborg, Sweden; 3 Centre for Mathematical Sciences, Mathematical Statistics, Lund University, Lund, Sweden; 4 Department of Energy Sciences, Faculty of Engineering, Lund University, Lund, Sweden; 5 Department of Clinical Sciences Lund, Anesthesia & Intensive Care, Skåne University Hospital, Lund University, Lund, Sweden; 6 Department of Clinical Sciences Lund, Neurology, Skåne University Hospital, Lund University, Lund, Sweden; 7 Department of Clinical Sciences Lund, Intensive and Perioperative Care, Skåne University Hospital, Lund University, Malmö, Sweden; 8 Department of Cardiology, The Heart Centre, Rigshospitalet University Hospital and Department of Clinical Medicine, University of Copenhagen, Copenhagen, Denmark; 9 Department of Critical Care, University Hospital of Wales, Cardiff, UK

**Keywords:** Machine learning, Artificial intelligence, Artificial neural networks, Out-of-hospital cardiac arrest, Cerebral performance category, Critical care, Intensive care, Prediction, Prognostication

## Abstract

**Background:**

Pre-hospital circumstances, cardiac arrest characteristics, comorbidities and clinical status on admission are strongly associated with outcome after out-of-hospital cardiac arrest (OHCA). Early prediction of outcome may inform prognosis, tailor therapy and help in interpreting the intervention effect in heterogenous clinical trials. This study aimed to create a model for early prediction of outcome by artificial neural networks (ANN) and use this model to investigate intervention effects on classes of illness severity in cardiac arrest patients treated with targeted temperature management (TTM).

**Methods:**

Using the cohort of the TTM trial, we performed a post hoc analysis of 932 unconscious patients from 36 centres with OHCA of a presumed cardiac cause. The patient outcome was the functional outcome, including survival at 180 days follow-up using a dichotomised Cerebral Performance Category (CPC) scale with good functional outcome defined as CPC 1–2 and poor functional outcome defined as CPC 3–5. Outcome prediction and severity class assignment were performed using a supervised machine learning model based on ANN.

**Results:**

The outcome was predicted with an area under the receiver operating characteristic curve (AUC) of 0.891 using 54 clinical variables available on admission to hospital, categorised as background, pre-hospital and admission data. Corresponding models using background, pre-hospital or admission variables separately had inferior prediction performance. When comparing the ANN model with a logistic regression-based model on the same cohort, the ANN model performed significantly better (*p* = 0.029). A simplified ANN model showed promising performance with an AUC above 0.852 when using three variables only: age, time to ROSC and first monitored rhythm. The ANN-stratified analyses showed similar intervention effect of TTM to 33 °C or 36 °C in predefined classes with different risk of a poor outcome.

**Conclusion:**

A supervised machine learning model using ANN predicted neurological recovery, including survival excellently, and outperformed a conventional model based on logistic regression. Among the data available at the time of hospitalisation, factors related to the pre-hospital setting carried most information. ANN may be used to stratify a heterogenous trial population in risk classes and help determine intervention effects across subgroups.

## Introduction

During the last decade, increased computational power and improved algorithms have led to a renaissance for machine learning as an alternative to traditional regression models to analyse large data sets. Machine learning has been found valuable in various clinical settings such as interpretation of ECG (electrocardiography) patterns and detection of cardiac arrest in emergency calls or in the emergency department, to predict outcome in traumatic brain injury and to predict the need for critical care as an alternative to conventional triage and early warning scores [1–5]. It has also been suggested for mortality prediction in patients admitted to intensive care units (ICUs) [6].

Recently, machine learning models have been used to predict the outcome in out-of-hospital cardiac arrest (OHCA) cohorts with high accuracy early in the chain of resuscitation, where overall mortality is above 80% [7, 8], but these models are not applicable to patients admitted to ICUs after OHCA. Several factors are known to influence the overall outcome in the OHCA population, including patients’ age and comorbidities, cardiac arrest characteristics and status on admission [9–16]. Albeit carrying important individual information, none of these variables is taken into account in the current recommended multimodal neurological prognostication algorithm [17, 18] as the independent prediction ability in each of these variables is limited. A number of prediction models have been developed using clinical variables available on hospital admission. Risk scores using logistic regression have been proposed and typically show moderate to good accuracy including the “CAHP (Cardiac Arrest Hospital Prognosis) risk score” [19], the “OHCA risk score” [20] and a scoring system published by Aschauer et al. in 2014 [21]. So far, none of these models has been precise enough to be used for individual prediction after OHCA. The “TTM risk score” based on data from the Target Temperature Management (TTM) trial, using ten independent predictors associated with a poor outcome including death at 6 months after OHCA, managed to achieve excellent discrimination of outcome with an area under the receiver operating characteristic curve (AUC) of 0.818–0.842 [22]. With robust and accurate algorithms for early classification of illness severity and mortality risk, multimodal prognostication could hopefully be further improved to tailor patients to individual therapy and intervention effects. This may possibly only be applicable to subgroups of patients which could be differentiated in future heterogeneous clinical trials.

Using the database from the TTM trial, we aimed to investigate whether an artificial neural network (ANN)—a supervised machine learning algorithm—could detect more complex dependencies between clinical variables available at hospital admission in OHCA survivors and perform early and reliable predictions of long-term functional outcome with even better accuracy than traditional regression models. We also wanted to investigate which part of the “chain of survival” contained the most predictive information based on background, pre-hospital and admission-centred data. Finally, an attempt was made to demonstrate any difference in treatment effect across risk classes of illness severity in the TTM trial.

## Materials and methods

### Study setting

We included all 939 patients enrolled in the TTM trial from 2010 to 2013 in 36 ICUs in Europe and Australia. The trial included comatose (Glasgow Coma Scale (GCS) ≤ 8) adults (≥ 18 years of age) with a sustained return of spontaneous circulation (ROSC) after successful resuscitation from OHCA of presumed cardiac cause. Patients were admitted to ICUs and randomised to TTM at 33 °C or 36 °C [23]. The trial protocol was approved by ethical committees in each participating country, and informed consent was waived or obtained from all participants or relatives according to national legislation, in line with the Helsinki Declaration [24]. Patient data were entered in an online electronic case record form and externally monitored. The results of the main trial were subjected to sensitivity analyses for time, study centre and other possible biases and have been elaborated in post hoc analyses and substudies. All have shown similar outcomes in both temperature groups [25–28]. Therefore, the pooled TTM data set was used for the present analysis.

### Variables

Baseline comorbidities, demographics, pre-hospital data, arrest characteristics and physiological variables, as well as admission data, were systematically collected according to the Utstein criteria [29, 30] and categorised as background-, pre-hospital and admission variables (Table 1). Time from cardiac arrest (CA) to initiation of basic life support (BLS; administered by bystanders or first responders) and advanced life support (ALS) was recorded. No-flow and low-flow times were defined as the time from CA to the start of CPR (BLS or ALS) and the time from the start of CPR to ROSC, respectively. Time to ROSC was defined as the time from CA to the first recorded time point of sustained (> 20 min) spontaneous circulation. “No flow” (indicating the time from arrest until the start of cardiopulmonary resuscitation (CPR)) and “low flow” (indicating the time from the start of CPR until the return of spontaneous circulation (ROSC)) are often used to describe the circumstances of the CPR treatment. However, from a clinical point of view, these terms are less intuitive compared to “bystander CPR”, “time to advanced CPR” and “time to ROSC”; therefore, two data sets were created. Data set A—all variables plus “bystander CPR”, “time to advanced CPR” and “time to ROSC”, but not “no flow” and “low flow”. Data set B—all variables plus “no flow” and “low flow”, but not “bystander CPR”, “time to advanced CPR” and “time to ROSC” (Table 1).

**Table 1:** Baseline characteristics stratified into good outcome (CPC 1–2) and poor outcome (CPC3–5) after 6 months

	CPC score 1–2	CPC score 3–5	***p*** value	Missing (%)
**Background**				
No. of patients	440	492		
Age, years (IQR)	61 (52–69)	68 (61–76)	< 0.001	0.0
Female sex (%)	66 (15.9)	111 (22.6)	0.004	0.0
Length, cm (IQR)	179 (171–183)	175 (167–180)	< 0.001	2.4
Weight, kg (IQR)	80 (73–90)	80 (70–90)	0.207	1.4
Chronic heart failure (%)	16 (3.6)	44 (9.0)	0.001	0.2
Previous myocardial infarction (%)	79 (18.0)	112 (22.8)	0.080	0.1
Ischaemic heart disease (%)	101 (23.0)	157 (32.0)	0.003	0.2
Previous cardiac arrhythmia (%)	60 (13.6)	103 (21.0)	0.004	0.1
Previous cardiac arrest (%)	9 (2.0)	12 (2.4)	0.851	0.1
Arterial hypertension (%)	150 (34.2)	222 (45.3)	0.001	0.3
TIA or stroke (%)	23 (5.2)	50 (10.2)	0.007	0.3
Epilepsy (%)	11 (2.5)	5 (1.0)	0.135	0.1
Diabetes (%)	51 (11.6)	89 (18.2)	0.007	0.5
Asthma or COPD (%)	31 (7.0)	65 (13.2)	0.003	0.0
Dialysis (%)	2 (0.5)	4 (0.8)	0.785	0.0
Haematological malignancy (%)	2 (0.5)	7 (1.4)	0.241	0.6
Other malignancies (%)	7 (1.6)	16 (3.3)	0.156	0.4
Alcoholism (%)	10 (2.3)	26 (5.3)	0.027	0.1
Previous PCI (%)	45 (10.2)	62 (12.7)	0.286	0.3
Previous CABG (%)	26 (5.9)	62 (12.7)	0.001	0.3
Previous valvular surgery (%)	10 (2.3)	15 (3.1)	0.590	0.4
Implantable cardioverter-defibrillator (%)	1 (0.2)	4 (0.8)	0.444	0.3
Pacemaker (%)	11 (2.5)	21 (4.3)	0.196	0.3
**Pre-hospital**				
Cardiac arrest location (%)			< 0.001	0.0
Place of residence	192 (43.6)	306 (62.2)		
Public place	216 (49.1)	166 (33.7)		
Others	32 (7.3)	20 (4.1)		
Bystander witnessed arrest (%)	406 (92.3)	427 (86.8)	0.009	0.0
Bystander defibrillation (%)	55 (12.5)	34 (6.9)	0.005	0.1
First monitored rhythm (%)			< 0.001	0.0
Non-perfusing ventricular tachycardia (VT)	11 (2.5)	12 (2.4)		
Ventricular fibrillation (VF)	391 (88.9)	311 (63.2)		
Asystole	12 (2.7)	100 (20.3)		
Pulseless electrical activity (PEA)	12 (2.7)	53 (10.8)		
Unknown	4 (0.9)	14 (2.8)		
ROSC after bystander defibrillation	10 (2.3)	2 (0.4)		
First rhythm shockable (%)	414 (94.1)	333 (67.7)	< 0.001	0.0
Automatic compression-decompression (%)			0.145	0.2
No	348 (79.1)	363 (74.1)		
Yes, manual	30 (6.8)	35 (7.1)		
Yes, mechanical	62 (14.1)	92 (18.8)		
Number of defibrillations (IQR)	3 (1–4)	2 (1–3)	0.001	0.5
Pre-hospital intubation (%)	273 (62.9)	352 (72.6)	0.002	1.0
Seizures before admission (%)			0.005	0.2
No	406 (92.5)	468 (95.3)		
Yes, before CA	21 (4.8)	6 (1.2)		
Yes, after resuscitation	12 (2.7)	17 (3.5)		
Total dose of adrenaline, mg (IQR)	1 (0–3)	3 (1–5)	< 0.001	0.4
**Data set A**				
CA to ALS, min (IQR)	8.00 (5.00–11.00)	10.00 (7.00–15.00)	< 0.001	1.5
CA to ROSC, min (IQR)	20.00 (14.75–30.00)	31.00 (21.00–47.00)	< 0.001	0.0
Bystander CPR (%)	347 (78.9)	331 (67.4)	< 0.001	0.1
**Data set B**				
No flow, min (IQR)^a^	1.00 (0.00–3.00)	2.00 (0.00–8.00)	< 0.001	0.5
Low flow, min (IQR)^b^	19.00 (12.00–27.00)	27.00 (17.00–40.25)	< 0.001	0.0
**Admission**				
Initial temperature, °C (IQR)	35.5 (34.9–36.0)	35.3 (34.4–36.0)	0.002	3.6
Glasgow Coma Scale (GCS) motor score = 1 (%)	173 (39.4)	316 (64.9)	< 0.001	0.6
Acute ST-infarction or LBBB	217 (49.5)	220 (45.4)	0.228	1.0
Blood glucose, mmol/L (IQR)	12.35 (9.47–16.00)	14.00 (10.60–18.00)	< 0.001	5.5
pO_2_, kPa (IQR)	18.3 (11.7–30.1)	18.9 (12.1–37.1)	0.344	7.4
pCO_2_, kPa (IQR)	6.0 (5.2–6.8)	6.3 (5.2–7.8)	0.003	5.8
Base excess, BE (IQR)	− 6.0 (− 10.0–4.0)	− 10.0 (− 14.5–5.0)	< 0.001	7.0
Potassium, mmol/L (IQR)	3.7 (3.4–4.2)	4.0 (3.5–4.5)	< 0.001	2.9
FiO_2_, % (IQR)	80 (50–100)	90 (53–100)	0.215	3.2
Creatinine, μmol/L (IQR)	95 (80–115)	115 (90–140)	< 0.001	3.1
Platelets, cells × 10^9^/L (IQR)	220 (185–265)	215 (170–274)	0.128	3.1
WBC, cells × 10^9^/L (IQR)	14.0 (10.6–18.0)	14.0 (10.4–18.5)	0.872	4.1
Cough reflex (%)	277 (70.1)	211 (48.6)	< 0.001	11.1
Spontaneous breathing (%)	310 (72.9)	284 (60.3)	< 0.001	3.9
pH (IQR)	7.27 (7.17–7.32)	7.19 (7.05–7.28)	< 0.001	4.6
Lactate, mmol/L (IQR)	4.6 (2.4–8.1)	6.9 (3.9–10.6)	< 0.001	6.3
Shock on admission (%)^c^	36 (8.2)	100 (20.3)	< 0.001	0.0
Pupil or corneal response (%)	392 (90.1)	327 (68.7)	< 0.001	2.3

Data are presented as *n* (%) or median (IQR). *n* denotes the number of cases with valid data. A *p* value of < 0.05 was considered significant. The 54 variables are grouped into background, pre-hospital and admission variables

*IQR* interquartile range, *CPC* cerebral performance category, *TIA* transient ischaemic attack, *COPD* chronic obstructive pulmonary disease, *PCI* percutaneous coronary intervention, *CABG* coronary artery bypass grafting, *VT* ventricular tachycardia, *VF* ventricular fibrillation, *PEA* pulseless electric activity, *ROSC* return of spontaneous circulation, *CA* cardiac arrest, *ALS* advanced life support, *CPR* cardiopulmonary resuscitation, *GCS* Glasgow Coma Scale, *LBBB* left bundle branch block, *WBC* white blood cell

^a^No flow is defined as the time from the arrest to the start of CPR

^b^Low flow is defined as the time from the start of CPR to ROSC

^c^Shock on admission is defined as systolic blood pressure of less than 90 mmHg for more than 30 min or end-organ hypoperfusion unless vasoactive drugs are administered

### Outcome

The main outcome of this study was 180 days functional outcome including survival using a dichotomised Cerebral Performance Category (CPC) scale where CPC 1–2 was categorised as a good functional outcome and CPC 3–5 as a poor functional outcome [31]. A good functional outcome (CPC 1–2) includes patients independent for daily activities but may have a minor disability. A poor functional outcome (CPC 3–5) includes patients dependent on others, in a coma or vegetative state and dead [32]. The CPC was graded at follow-up by a blinded assessor during a structured interview face-to-face or by telephone [33].

### Prediction models

We aimed to create two different predictions models: the best possible prediction model, which included 54 available input variables on patient admission to intensive care (Table 2), and a simplified prediction model by ranking all the variables after their individual performance adding one variable at the time according to their relative importance. The ranking of these variables was calculated by their individual effect on the AUC when subtracted from the overall model. We wanted to investigate how well our model performed compared to an earlier risk-scoring system based on logistic regression analysis of the same cohort [22].

**Table 2:** Predictor ranking and prediction performance in data set A

Table 2a			Table 2b		
Rank	Predictor	AUC	No. of variables	AUC_**CV**_	AUC_**test**_
1	Age	0.8188 (± 0.0207)	1	0.708 (± 0.0286)	0.657
2	Time to ROSC	0.8285 (± 0.0256)	2	0.780 (± 0.0113)	0.799
3	First monitored rhythm	0.8319 (± 0.0217)	3	0.820 (± 0.0106)	0.852
4	Previous cardiac arrest	0.8324 (± 0.0238)	4	0.822 (± 0.0169)	0.861
5	GCS motor score	0.8335 (± 0.0225)	5	0.832 (± 0.0229)	0.863
6	Dose of adrenaline	0.8337 (± 0.0244)	6	0.839 (± 0.0170)	0.826
7	Creatinine	0.8342 (± 0.0221)	7	0.846 (± 0.0117)	0.837
8	Cardiac arrest location	0.8356 (± 0.0234)	8	0.854 (± 0.0119)	0.857
9	Previous AMI	0.8358 (± 0.0227)	9	0.843 (± 0.0129)	0.835
10	Diabetes	0.8358 (± 0.0221)	10	0.840 (± 0.0182)	0.844
11	Length	0.8358 (± 0.0176)	11	0.848 (± 0.0173)	0.869
12	Time to Advanced CPR	0.8360 (± 0.0189)	12	0.853 (± 0.0142)	0.870
13	pH	0.8363 (± 0.0260)	13	0.851 (± 0.0266)	0.880
14	Platelets	0.8363 (± 0.0234)	14	0.849 (± 0.0079)	0.875
15	Bystander witnessed arrest	0.8366 (± 0.0190)	15	0.852 (± 0.0188)	0.886
			**All 54**	**0.852 (± 0.0172)**	**0.891**

The ranking of the variables in the data set was calculated by their individual effect on the AUC when subtracted from the overall model. The AUC values in Table 2a represent how much the performance decreases when the corresponding variable is excluded from the model

The AUC values in Table 2b show how much the prediction performance of the model increase by adding one variable at the time (based on their relative importance in Table 2a) to the model. AUC_CV_ values represent the training set, and AUC_test_ the test set. When using all available variables at patient admission to intensive care, the AUC was 0.891, indicating an excellent performance of predicting long-term functional outcome

*AUC* area under the curve, *ROSC* return of spontaneous circulation, *GCS* Glasgow Coma Scale, *AMI* acute myocardial infarction, *CPR* cardiopulmonary resuscitation

We also wanted to analyse which variables and clinical information that carry the most predictive information among background, pre-hospital or admission variables and compare this with the overall model. Finally, we performed an analysis of the intervention effect of 33 °C vs 36 °C stratified to risk classes. The five risk classes were defined as 0–20%, 20–40%, 40–60%, 60–80% and 80–100% risk of a poor outcome at 180 days based on variables available at randomisation.

### Designing and evaluating the ANN

A test set, corresponding to 10% of the data, was randomly chosen and set aside to test the performance of the final ANN model. The remaining data (90%) was used for training. The training set was randomly divided into five equal-sized groups, to allow for cross-validation during model development. Missing values were imputed using a simple mean or mode substitution based on the training set.

**Fig. 1:**
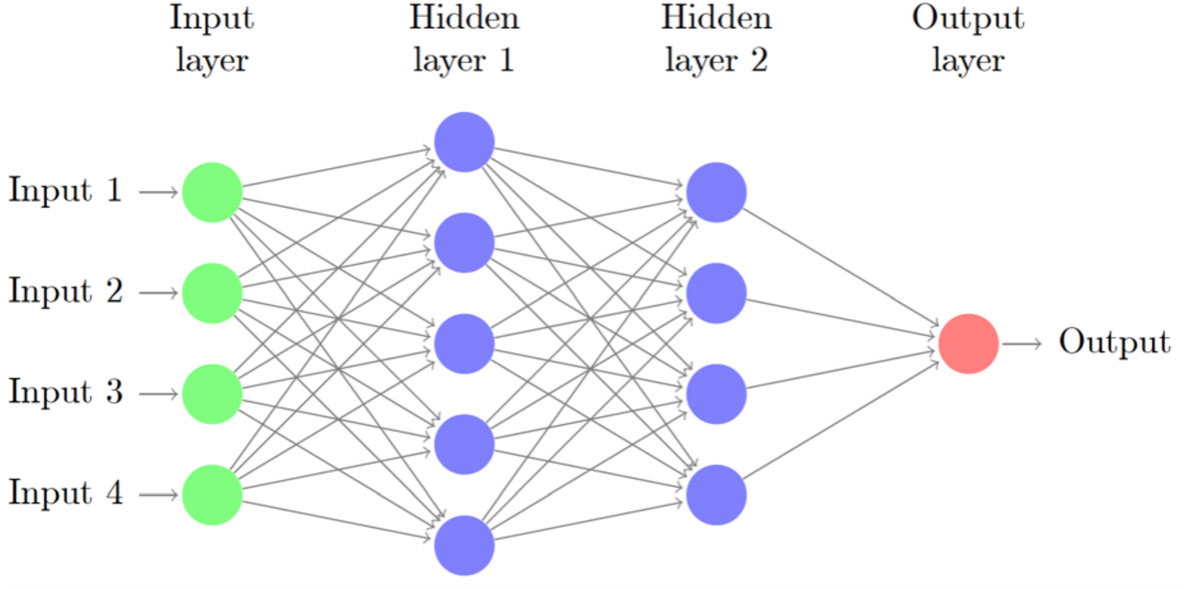
ANN, artificial neural network. A schematic ANN with one input layer, two hidden layers and one single output layer. All nodes in the network are connected in resemblance to the human central nervous system. The input layers in our ANN consisted of variables (background, pre-hospital and/or admission data) whereas the output layer was the outcome variable Cerebral Performance Category (CPC) scale dichotomised into good (CPC 1–2) or poor (CPC 3–5) functional outcome

Our ANN consisted of one input layer, a number of hidden layers and one output layer (Fig. 1). A Bayesian optimisation approach, based on the Tree-structured Parzen Estimator (TPE), was used to find the best possible network architecture [34]. The search for optimal hyperparameters was performed with the following limits: 1–4 hidden layers, 5–400 nodes in each layer, batch size between 1 and 128, and learning rate 10^−7^–1, and the activation function was chosen to be either to be the rectified linear unit (ReLU) or the hyperbolic tangent function. To improve generalisation, Bayesian optimisation was used to determine the most suitable regularisation parameters. The algorithm chose between the weight decay techniques L1−, L2−norm penalties distributed between 10−5 and 1 or max-norm regularisation distributed between one and five. To further improve generalisation, dropout [35] and batch normalisation was applied [36]. The probability of a node being dropped was uniformly distributed between 0 and 0.5 in the hidden layers, and 0 and 0.3 in the input layer. The sigmoid activation function was used for the single node in the output layer [37]. All networks were trained using early stopping, with patience of 50 epochs. The maximum number of epochs was set to 1000. Two different methods for optimising the loss function were tested: the Adam implementation of stochastic gradient descent (SGD) and a slightly different version called Adam AMSGrad [38]. The hyperparameters resulting in the best performing networks were as follows: a one-layer network with 149 nodes using the ReLU activation function and L2-norm weight decay with *λ* = 0.1374. The input dropout rate was 0.240, and the hidden dropout rate was 0.405. Furthermore, the optimisation algorithm was Adam AMSGrad, with a learning rate of 0.00197 and a batch size of 29. Batch normalisation was used. All networks were created using TensorFlow, an open-source machine learning framework developed by Google [39].

### Statistical analysis

All continuous variables were presented as median with upper and lower quartiles, the interquartile range (IQR). Categorical variables were presented as numbers and percentages. The fraction of missing data is reported in Table 1. For comparison between the groups, the Mann-Whitney *U* test was used for continuous data and Fisher’s exact test for categorical data. To evaluate the performance of the ANN model, we examined the receiver operating characteristics curves (ROC), which plots sensitivity against 1-specificity, for all threshold settings. We used the area under the curve (AUC) as a performance measure [40], and the method of DeLong et al. [41] was used for the calculation of AUC differences.

A forest plot was created to assess the association between five predefined ANN-stratified risk classes of a poor outcome and treatment with targeted temperature management at 33 °C and 36 °C. All *p* values were two-tailed, and a *p* < 0.05 was considered significant. We used the STROBE Statement style for the study manuscript [42].

## Results

Of the 939 patients enrolled in the TTM trial, 932 were included in our study for the final analysis. Six patients were excluded due to missing outcomes, and one patient was excluded due to a high number of missing values (> 40). The population characteristics were categorised and presented as background, pre-hospital and admission variables in Table 1. Good functional outcome (CPC 1–2) was found in 440 (47%) patients, and 492 (53%) patients had a poor functional outcome (CPC 3–5) at 180 days follow-up. Patients with poor functional outcome were significantly older (68 vs 61 years, *p* < 0.001), more often female (22.6% vs 15.0%, *p* < 0.01) and had a higher degree of cardiovascular comorbidity compared to patients with good functional outcome. Patients with a poor functional outcome also presented with worse clinical neurological findings, more metabolic and respiratory acidosis and the presence of circulatory shock on admission (Table 1).

**Fig. 2:**
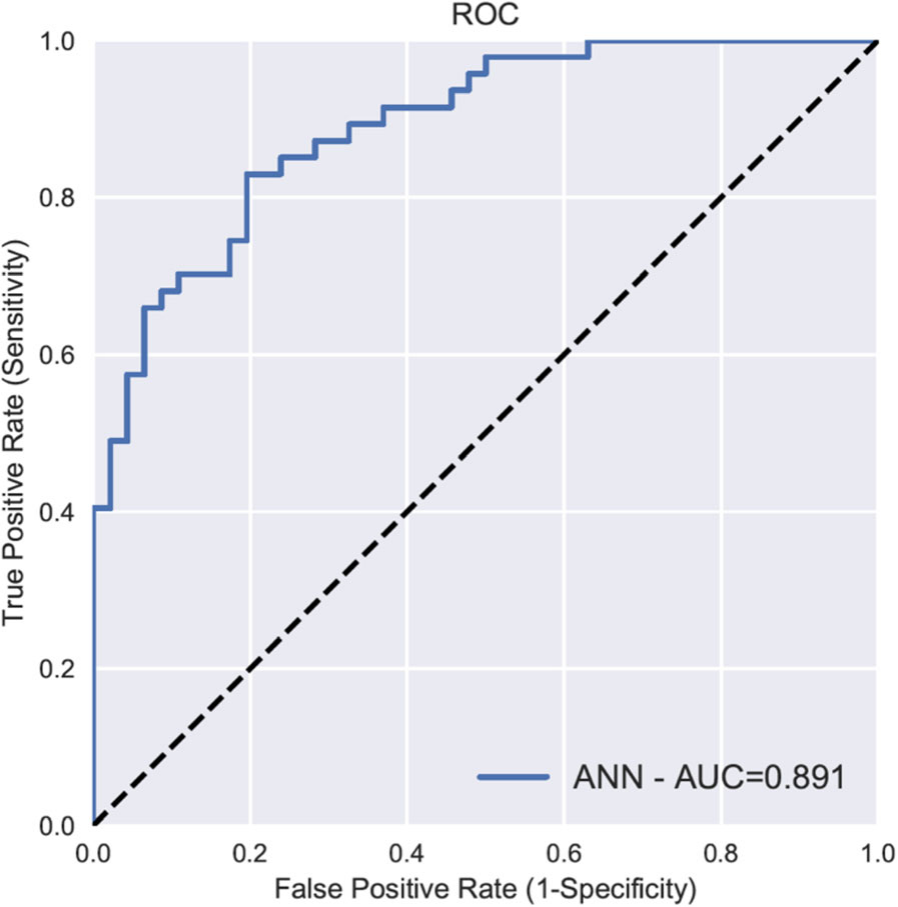
Prediction performance. The prediction performance of long-term functional outcome is expressed as AUC in a ROC curve, by an ANN model using all 54 variables available on admission to intensive care. Of the 932 patients included in the study, 93 patients (10%) was randomly chosen and removed from the training set on which the ANN algorithm trained its prediction model. The trained ANN was then used to make a prediction of the outcome on the 93 patients earlier removed to represent the test set. The mean AUC for our ANN was 0.891, indicating an excellent performance to predict long-term outcome. AUC, area under the curve; ROC, receiver operating characteristics; ANN, artificial neural network

The data set was then randomly divided into a training set for developing the ANN model (*n* = 839) and a test set (*n* = 93) for independent *performance measurement* of the model’s generalisability. The overall ANN model, based on the 54 variables of data set A, showed a good prognostic capability in predicting outcome after 6 months. The cross-validated AUC (from the training set) was 0.852 ± 0.017 (Table 2b), and the AUC on the independent internal validation data set (the test set, *n* = 93) was 0.891, as shown in Fig. 2. Similar results were found when using the 53 variables in data set B (cross-validated AUC 0.852 ± 0.018 and test set AUC 0.889). As the variables in data set A are more intuitive to use in a clinical setting, and the AUC was similar in both data sets, we chose to focus on data set A.

**Fig. 3:**
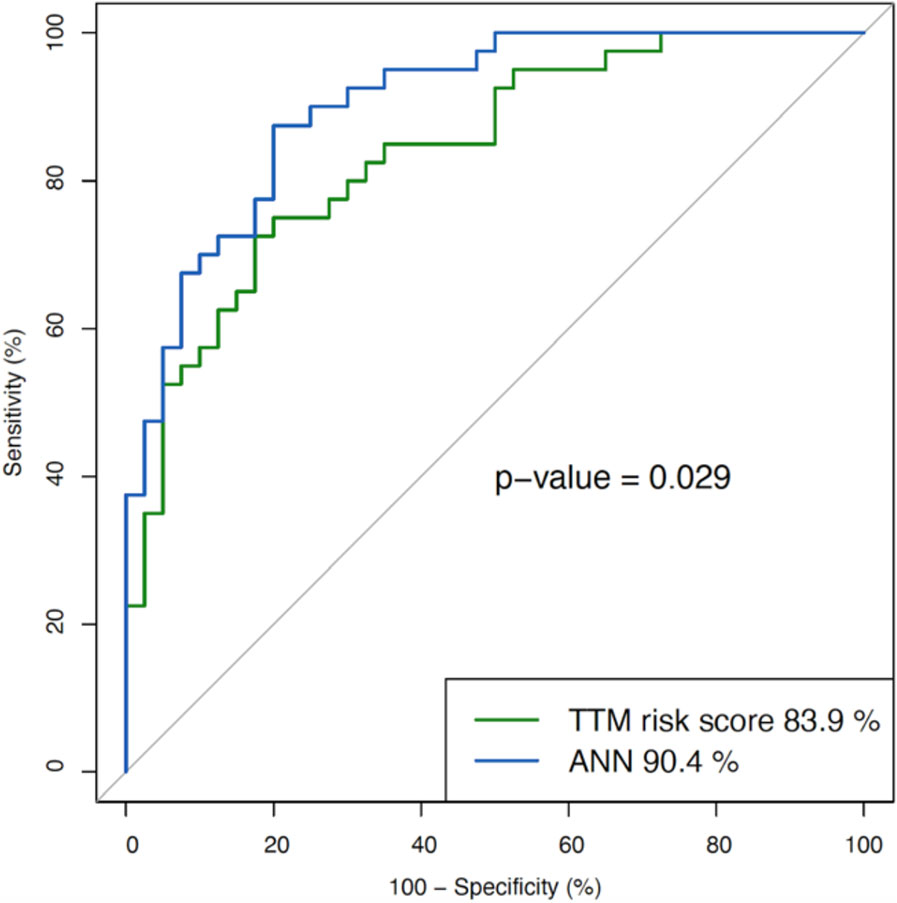
Prediction performance in comparison. Comparison of the prediction performance of long-term outcome expressed as AUC in ROC curves, between our ANN model (blue) and the TTM risk score (green) from Martinell et al. The ANN model (AUC = 0.904) outperformed the TTM risk score (AUC = 0.839) significantly (*p* = 0.029) in a comparative analysis based on 80 patients (test set) from the TTM data set. Since the “TTM risk score” does not have a strategy for handling missing values, 13 patients were removed from the original test set with 93 patients when comparing the two models. The ANN AUCs in Figs. 2 and 3 differ for the same reason. AUC, area under the curve; ROC, receiver operating characteristics; ANN, artificial neural network; TTM, targeted temperature management

When using information from the background, pre-hospital or admission variables in separate analyses, the model including only pre-hospital data performed best with an AUC of 0.861 on the validation set (test set) compared to admission data only (AUC test 0.784) or background data only (AUC test 0.670). When comparing the performance difference between the “TTM risk score” [22] and our ANN model on the test set, the ANN model had a significantly better AUC (0.904 vs 0.839, *p* = 0.029), as shown in Fig. 3.

**Fig. 4:**
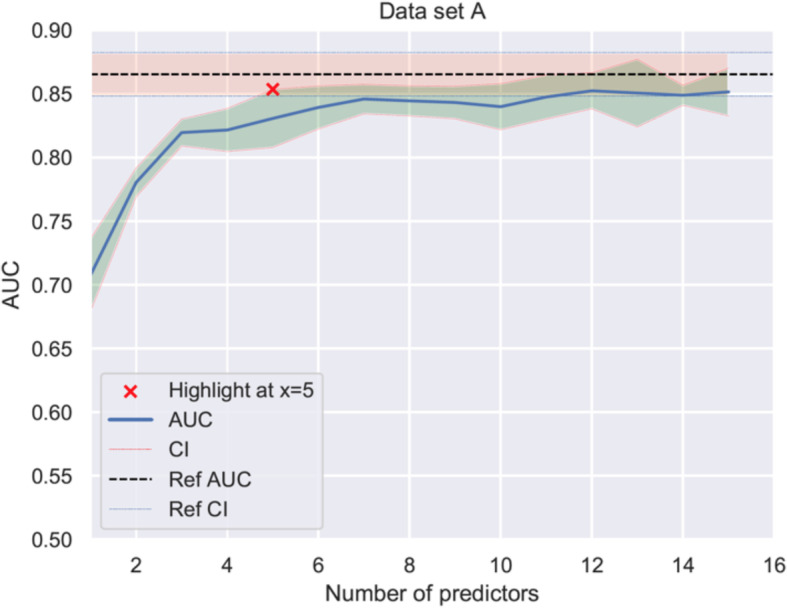
Increased prediction performance when adding variables. The change in AUC during training (AUC_CV_) when adding one predictor at the time and running the optimization process each time. The predictive performance of the model (represented by the blue line and its corresponding CI in green area) initially increased rapidly, but then levelled out, gradually approaching the reference AUC (represented by the dotted line and its corresponding CI in the pink area) of the model using all 54 variables. After adding five variables, there was no significant difference between the two models regarding prediction performance, marked by a red X in the figure. AUC, area under the curve; CI, confidence interval

**Fig. 5:**
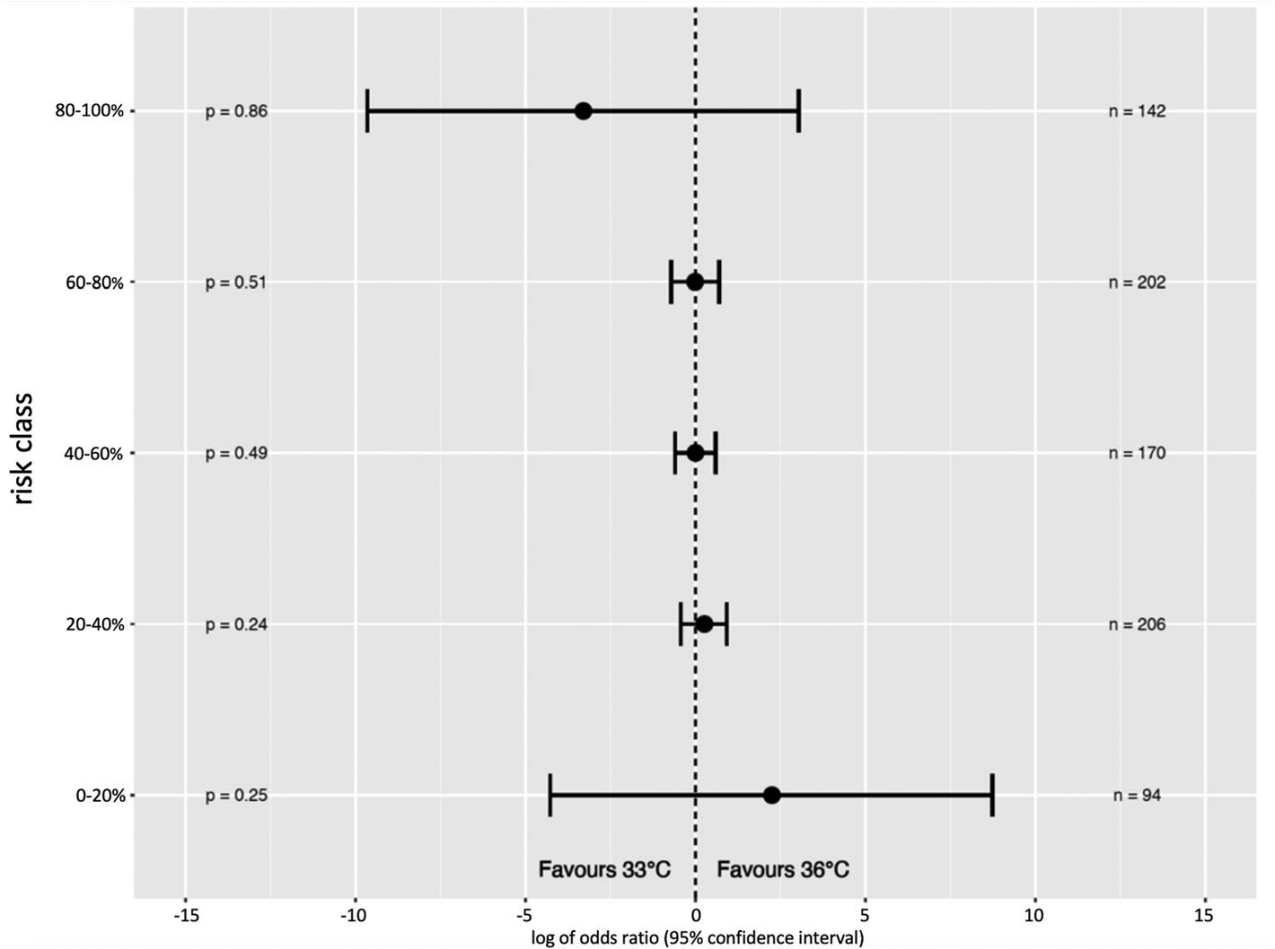
Diagnostic odds ratio for the artificial neural network (ANN)-stratified risk groups The forest plot shows the logarithmic diagnostic odds ratio for five ANN-stratified risk groups of CPC score > 2 and its association to treatment with targeted temperature management at 33 °C and 36 °C. A diagnostic odds ratio > 1 implies a better functional outcome when treated with 36 °C compared to 33 °C. CPC, cerebral performance category

To create a simplified prediction model, all 54 variables were ranked based on their individual importance and their effect on the AUC when removed from the model. The ranking for the 15 most important variables and the corresponding AUC, when adding them one at a time to the model, is shown in Table 2b. The predictive performance initially increased rapidly, but then levelled out, gradually approaching the value of the reference AUC of the model using all 54 variables (Fig. 4). After adding five variables, there was no further significant increase in performance between the models. Of all variables available at admission to hospital, “age”, “time to ROSC” and “first monitored rhythm” were the three variables carrying the most predictive information. When only these three variables were combined in a neural network model, they showed good discrimination with a cross-validated AUC of 0.820 ± 0.011 (training set) and an AUC of 0.852 on the validation test set (Table 2b). Finally, we divided the trial cohort into five classes of risk of a poor outcome. The ANN-stratified analyses showed similar treatment effect of TTM to 33 °C or 36 °C in these five predefined risk classes as measured by the logarithm of the diagnostic odds ratio (log (DOR)) (Fig. 5). Risk prediction in risk class 0–20% had a log (DOR) of 2.2 (*n* = 94, CI95% − 4.2–8.7, *p* = 0,25); in risk class 20–40%, a log (DOR) of 0.24 (*n* = 206, CI95% − 0.43–0.92, *p* = 0.24); in risk class 40–60%, a log (DOR) of 0.01 (*n* = 170, CI95% − 0.59–0.61, *p* = 0.49); in risk class 60–80%, a log (DOR) of 0 (*n* = 202, CI95% − 0.72–0.70, *p* = 0.51); and in risk class 80–100%, a log (DOR) of − 3.3 (*n* = 142, CI95% − 9.7–3, *p* = 0.85).

## Discussion

In this study, we performed a post hoc analysis of OHCA patients included in the TTM trial and used artificial neural network (ANN), a supervised machine learning model, to predict the functional outcome including survival at 180 days, with information readily available at the time of hospitalisation. Our model performed predicted outcome better compared to a corresponding logistic regression model in a prior study of the same cohort [22]. The overall ANN model, based on all 54 variables available on admission, showed an excellent capability of outcome prediction during the internal validation training and performed even better on the test set with an AUC of 0.891. Using only the three most important independent factors (age, time to ROSC and first monitored rhythm, which are variables readily known on arrival in the emergency room) in an ANN led to a model with an excellent predictive ability on the test set with an AUC of 0.852 which is better compared to most proposed models in the field [19–22]. To identify which type of information that carries the most valuable prediction of outcome, we also designed a model that used the three available data categories (background, pre-hospital and admission data) separately. This approach decreased the prediction capability compared to the overall model, but variables from the pre-hospital setting carried the most information.

Large pragmatic clinical trials have been criticised for being heterogeneous and possibly dilute any intervention effect that theoretically may be relevant for subgroups of patients [43]. In this study, we performed a stratified analysis using ANN to define risk classes in relation to the outcome where any intervention effect could be studied. Our models did not show any significant difference in the intervention effect of 33 °C or 36 °C regarding the outcome when dividing the TTM trial population into five different risk classes for a poor outcome. The intervention effect was thus uniform across the risk classes, which strengthens the main conclusion of the trial, but also suggest a possible model for detection of subgroup effect in other clinical trials.

A number of attempts have been made to create robust and straight-forward outcome prediction scores in the OHCA population at admission to intensive care, in order to early identify patients with a significant risk of a poor outcome and stratify the severity of illness better than traditional classifications as the Acute Physiology, Age and Chronic Health Evaluation (APACHE) and Simplified Acute Physiology Score (SAPS) known to underperform in OHCA populations [19–21, 44]. An interesting future use of ANN algorithms would be the possibility to reliably assess individual risk of a poor outcome in OHCA patients which could have clinical implications for early allocation to specific interventions (tailored therapy) and later in the clinical course to inform prognosis and continued life support. In recent years, machine learning has been used increasingly in various studies and proved to be a promising method for data analyses. Machine learning has advantages compared to traditional regression models, i.e. the ability to detect correlations between independent variables in large complex data sets and to find trends or patterns in subsets of data. Recently published studies have shown the potential of machine learning regarding OHCA prediction with very good performance [7, 8]. In a study from Kwon et al., over 36,000 OHCA patients were included, and a deep learning-based OHCA prognostic system showed an impressive performance to predict neurologic recovery and survival to discharge of OHCA patients, with an AUC of 0.953 ± 0.001. However, no information regarding the long-term outcome in these patients was presented, and the overall mortality was very high, inherently increasing the possibility to reach high AUCs. The cohort used in the study was heterogeneous including more than 8000 patients (22%) with cardiac arrest of a traumatic cause, known to have a poor outcome and therefore probably contributing significantly to the predictive performance of the models [8]. In a population with about 50% survival, as for OHCA patients admitted to intensive care, our model reaching an AUC close to around 0.9 using early data alone should encourage validation in separate and prospective cohorts. There have been some studies indicating the lack of machine learning performance benefits over logistic regression-based models. In a systematic review of Christodoulou et al. from 2019, no evidence of performance superiority of machine learning over logistic regression was found [45]. The study did, however, conclude that improvements in both methodology and reporting are needed for trials that compare modelling algorithms, and our study indeed indicated a significantly better performance with ANN compared to the state-of-the-art logistic regression.

There are a number of limitations to this study. The majority of the variables had missing values, which leads to a number of challenges when developing a prediction model. To ensure that not too many patients or too many important variables were removed from the data set, we chose a simple strategy to replace them by mean values for continuous variables and mode values for categorical variables. Data collected in the pre-hospital setting might be imprecise due to the challenge of registering exact and valid information in that situation. Moreover, the TTM trial cohort is a selected population, including only patients with a presumed cardiac cause of cardiac arrest, making it difficult to generalise our results to unselected cardiac arrest patients. There are discrepancies between the cross-validating (training) AUC and the resulting AUC from the test set. This is normal [46], but the fact that the models performed better on the test sets is, however, noteworthy. Due to the nature of ANNs, there are two likely factors that play a major role: the number of patients in the test set and the fact that we were using the ensemble of networks created during cross-validation to make predictions on the test set. The ensemble technique is a widely used regularisation method. Employing it should result, as in this case based on 5-fold cross-validation, in an increased generalisability of the model. Finally, data analysis using ANN models is still somewhat of a “black box” when it comes to applying the results to a real-life clinical setting due to the complexity of biology and the variable medical contexts.

Study strengths include the use of a well-defined cohort of OHCA patients. The TTM trial was an international multicentre randomised controlled trial with predefined protocol-based criteria for inclusion and treatment. There were strict rules for multimodal neurological prognostication and withdrawal of life-sustaining therapy. The long-term follow-up on outcome was performed with minimal data loss and assessed by a blinded assessor at a meeting with the patient and the patient’s next-of-kin according to a structured protocol, including neurological examinations and face-to-face interviews.

Robust and straight-forward prediction scores used as a practical decision tool to support clinical assessments would probably improve the overall cardiac arrest care by directing very advanced and potentially high-risk invasive treatment to those patients who may benefit from it. Such scores would hopefully also increase the ability to provide reliable prognostic information to next-of-kin, earlier than the observation time of at least 72 h, which is the current recommendation for neurological prognostication after cardiac arrest [17, 18, 47, 48].

We believe that this study is an important step towards improved outcome prediction in comatose patients surviving cardiac arrest with a good functional outcome. In the near future, we will have the results from the TTM2 trial with 1900 patients [49], offering the use of an even larger unique registry with OHCA patients for ANN analyses and hopefully improving the outcome prediction in these patients. There are some obvious medical and ethical implications as well as resource aspects that may benefit from the progression of future reliable cardiac arrest-specific severity scores for early outcome prediction. Future studies should investigate if outcome prediction performance increases significantly by adding additional data and clinical variables such as early electroencephalography, neuroimaging and biomarkers. Finally, to be able to detect subgroups in an OHCA population with an increased risk of a poor outcome or subgroups that may benefit from a specific intervention or need extensive rehabilitation, further studies on larger data sets are necessary to demonstrate significant associations.

## Conclusion

Our supervised machine learning model of ANN predicted neurological recovery, including survival excellently and outperformed a conventional model based on logistic regression. By data available at time of hospitalisation, factors related to the pre-hospital setting carried the most predictive information. ANN may stratify a heterogenous trial population in risk classes and help determine intervention effect across subgroups.

## Data Availability

The data is available from the Target Temperature Management trial steering group after an approval process.

## Abbreviations

ALS: Advanced life support
AMI: Acute myocardial infarction
ANN: Artificial neural network
APACHE: The Acute Physiology, Age and Chronic Health Evaluation
AUC: Area under the curve
BLS: Basic life support
CA: Cardiac arrest
CABG: Coronary bypass grafting
CAHP: Cardiac Arrest Hospital Prognosis
CI: Confidence intervals
COPD: Chronic obstructive pulmonary disease
CPC: Cerebral Performance Category
CPR: Cardiopulmonary resuscitation
ECG: Electrocardiography
GCS: Glasgow Coma Scale
ICU: Intensive care unit
IQR: Interquartile range
LBBB: Left bundle branch block
OHCA: Out-of-hospital cardiac arrest
PCI: Percutaneous coronary intervention
PEA: Pulseless electric activity
ReLU: Rectified linear unit
ROC: Receiver operating characteristics
ROSC: Return of spontaneous circulation
SAPS: Simplified Acute Physiology Score
SGD: Stochastic gradient descent
STROBE: Strengthening the Reporting of Observational Studies in Epidemiology
TIA: Transient ischaemic attack
TPE: Tree-structured Parzen Estimator
TTM: Targeted temperature management
VF: Ventricular fibrillation
VT: Ventricular tachycardia
WBC: White blood cell
